# Enhanced optical encryption via polarization-dependent multi-channel metasurfaces

**DOI:** 10.1515/nanoph-2024-0746

**Published:** 2025-02-13

**Authors:** Minghao Ning, Haozong Zhong, Zhen Gu, Ling-En Zhang, Ning Qu, Jun Ding, Tao Li, Lin Li

**Affiliations:** State Key Laboratory of Precision Spectroscopy, 12655 East China Normal University, Shanghai 200241, China; Shanghai Key Laboratory of Multidimensional Information Processing, Key Laboratory of Polar Materials and Devices, 12655 East China Normal University, Shanghai 200241, China; National Laboratory of Solid State Microstructures, College of Engineering and Applied Sciences, Nanjing University, Nanjing 210093, China; State Key Laboratory of Precision Spectroscopy, School of Physics and Electronic Science, 12655 East China Normal University, Shanghai 200241, China; Collaborative Innovation Center of Extreme Optics, Shanxi University, Taiyuan 030006, China

**Keywords:** optical encryption, metasurface, polarization-dependent multi-channel, VSS encryption

## Abstract

Optical encryption offers a powerful platform for secure information transfer, combining low power consumption, high-speed transmission, and intuitive visualization. Metasurfaces, with their unprecedented ability to manipulate light across multiple degrees of freedom within quasi-two-dimensional nanostructures, are emerging as promising devices for advanced encryption. However, encryption capacity remains constrained by limited information channels. Here, we present a visual secret sharing (VSS) scheme utilizing metasurfaces with multiple polarization-dependent channels and minimized crosstalk. Using a global optimization strategy for nanostructure geometries across the entire metasurface, we successfully realize eight independent polarization channels with negligible crosstalk. By encoding both the key and information into these channels with a modified VSS scheme, we demonstrate the complete recovery of seven plaintexts. This strategy supports scalable, high-capacity encryption, and can incorporate additional optical degrees of freedom, offering advanced solutions for advanced secure communication, information storage, and anti-counterfeiting.

## 1 Introduction

Information security is essential for protecting secure communications, privacy, and various aspects of daily life. A number of modern encryption techniques have been developed to prevent unauthorized decryption or data theft. Optical encryption, with its advantages of low power consumption, multi-dimensional capabilities, and parallel processing, provides an important platform for secure data transmission and has found widespread applications in secure communication [1], [2], [3], [4], [5], [6], [7], [8], [9], [10], [11]. Over the past few decades, optical encryption technologies have made significant progress, including optical watermarking [12], [13], steganography [14], and visual cryptography [15], [16]. Among these methods, visual secret sharing (VSS) stands out as a notable encryption technique that divides a secret pixel into multiple shares and the original information can only be reconstructed when a sufficient number of these shares are combined [17], [18], [19], [20], [21]. The number of encryption channels is essential to both of the information capacity and the security strength of such schemes. However, traditional VSS methods face inherent limitations in increasing the number of encryption channels. Meanwhile, it is challenge in complete recovery of the original information after decryption to the traditional methods.

In the realm of optical encryption, holography has emerged as a pivotal technology for counterfeiting prevention, with applications in identity verification, travel documents, and currency security. However, conventional optical systems often suffer from bulky device sizes, limiting their practicality in compact applications. In contrast, metasurfaces, composed of well-designed nanostructures, offer unprecedented control over multiple optical degrees of freedom at the subwavelength scale, such as polarization [22], [23], phase [24], [25], wavelength [26], [27], [28], [29], [30], angular momentum [31], [32], [33], [34], among others. This exceptional ability to manipulate light offers new opportunities for developing extended and well-controlled optical information channels [35], [36], [37], [38], [39], [40], [41], [42]. These properties make metasurfaces particularly well-suited for advanced optical applications requiring both high precision and miniaturization [43], [44], [45], [46], [47], [48], [49], [50], [51], [52], [53], [54], [55], [56], [57], [58], [59], [60], [61]. Recently, metasurface-based holography has emerged as a powerful and versatile approach for optical encryption [1], [7], [8], [9], [62], [63], [64]. By encoding information into metasurfaces, data can be accessed and decrypted only through pre-defined, sophisticated optical parameters. Despite these advances, the full potential of metasurface multiplexing to enhance information capacity and strengthen encryption security remains largely unexplored.

In this work, we demonstrate an enhanced secret sharing encryption scheme based on multiple polarization-dependent channels with a metasurface. To increase information capacity and strengthen encryption security, eight independent polarization-dependent channels with minimal crosstalk are achieved by utilizing a global optimization of the nanostructure geometries across the overall metasurface. Both the encryption key and the information are encoded to these eight channels, enabling the complete recovery of seven distinct images. Additionally, to further enhance the accuracy of the encryption, we incorporate a VSS strategy using BITXOR logic, addressing the issues of low fidelity of the decrypted information in conventional VSS techniques and achieving 100 % fidelity. Compared to previous metasurface-based polarization dependent holographic encryption methods, this approach offers a larger number of encryption channels, reduced channel crosstalk, and enhance the accuracy of the decrypted information. Moreover, this approach can be integrated with other optical degrees of freedom to further expand the number of information channels and overall capacity.

## 2 Implementation process of enhanced VSS encryption scheme via polarization-dependent multi-channel metasurfaces

**Figure 1:**
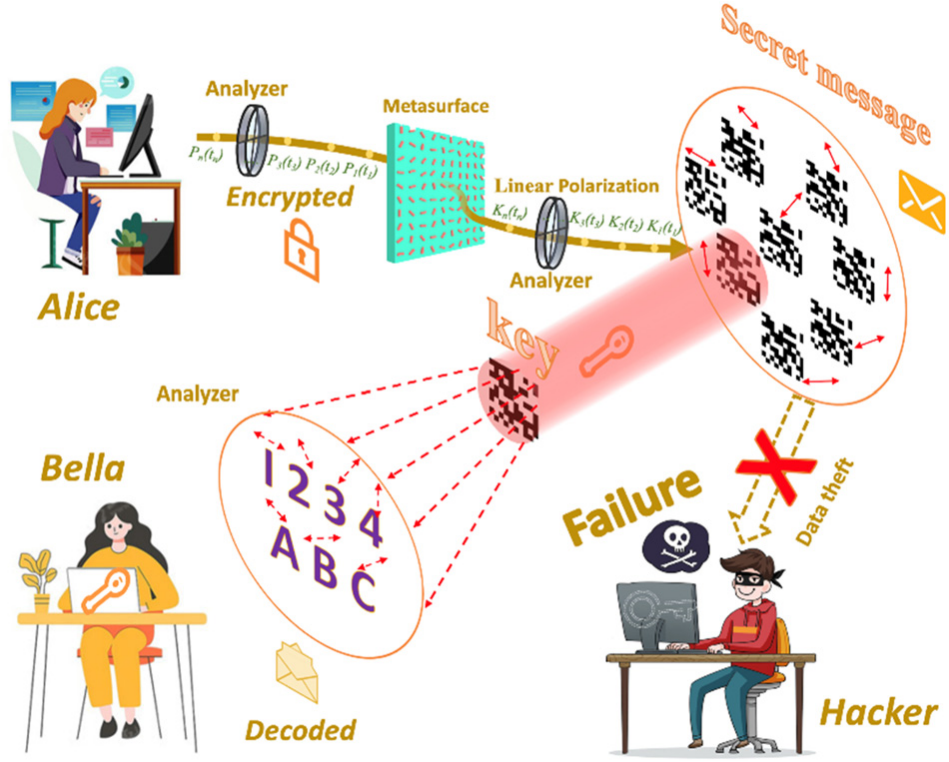
The polarization-dependent multi-channel encryption and decryption process with metasurface.

Figure 1 illustrates the process of secure information encryption and transmission between users utilizing polarization-dependent multi-channel metasurfaces. In this scenario, Alice aims to send a message to Bella. To ensure secure and accurate transmission, Alice employs a polarization-dependent multi-channel metasurface to encrypt the image information using a private key. Both the information and the private key are encoded into polarization-dependent channels. This encryption stratagem is based on a VSS scheme, where the original information can only be recovered by precisely analyzing the polarization, being aware of the private key, and employing the correct VSS algorithm. During communication, Alice sequentially encodes the encrypted information and key into the polarization-dependent channels, denoted as { $P_{1}\left(t_{1}\right)P_{2}\left(t_{2}\right)P_{3}\left(t_{3}\right)\ldots P_{n}\left(t_{n}\right)$ }, by adjusting the polarizer. Bella then receives the holographically encrypted information from each polarization channel and, using the private key and the correct VSS algorithm, successfully decrypts the original image data. If an incorrect polarization channel is selected or the private key and encryption logic are unknown, the plaintext information cannot be recovered (e.g., the image of the hacker), thereby significantly enhancing the encryption system’s security. Meanwhile, the information capacity could also be greatly increased by employing more multiplexing channels.

**Figure 2:**
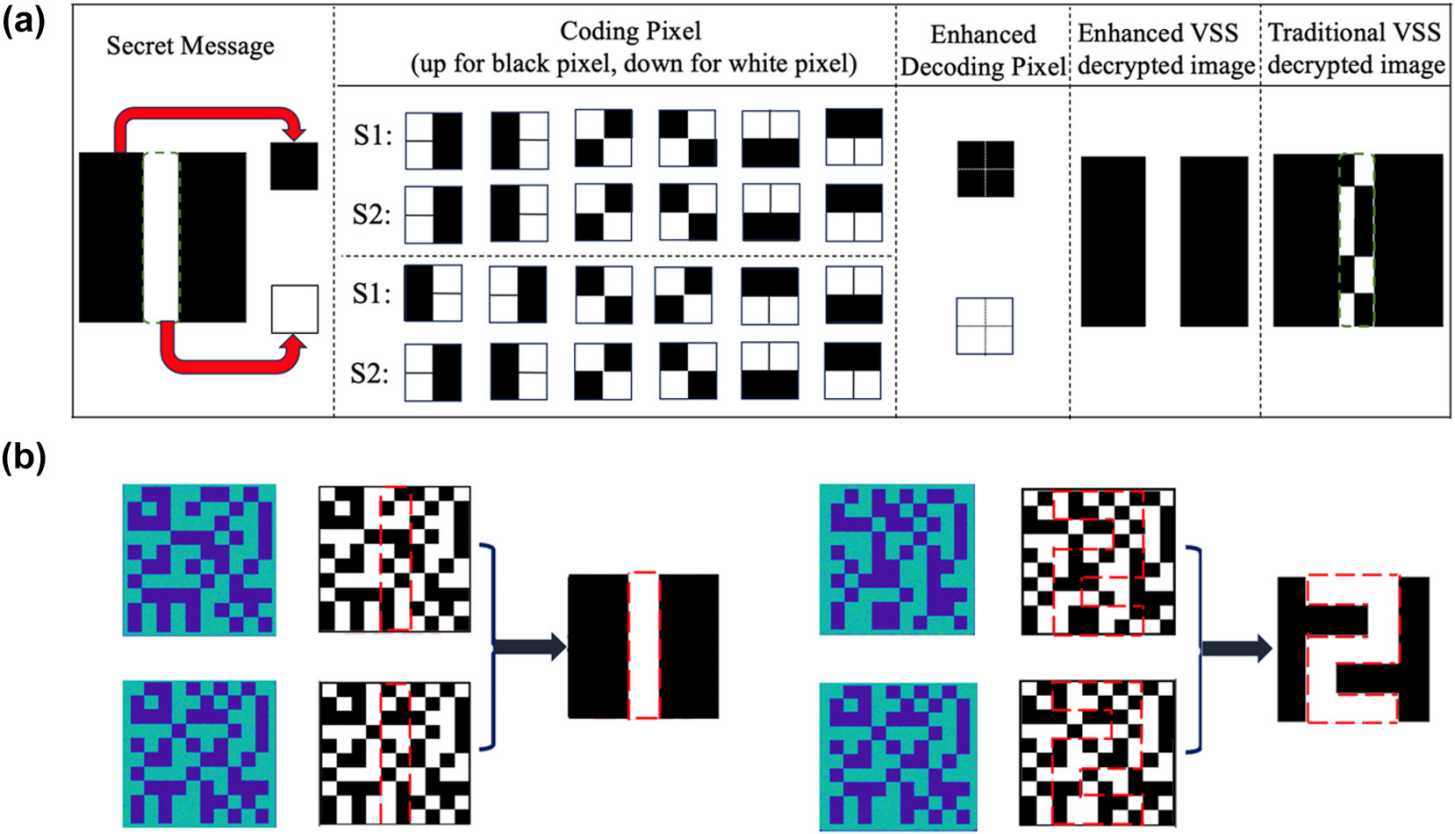
Enhanced VSS encryption method. (a) Encryption framework of the enhanced VSS encoding. Encoding of corresponding pixels in secret message in both the traditional and the enhanced (2, 2) VSS encryption methods. (Black pixels represent 0, while white pixels represent 1 and detailed explanations of VSS encryption are provided in the supplement.) (b) The numerical simulation of the encrypted hologram images and the corresponding decryption results.

Figure 2(a) shows the enhanced VSS encoding process. In this approach, black and white pixels in the plaintext are encoded to generate corresponding secret keys (SKs). In conventional VSS scheme (the detailed description is provided in the supplement), ‘AND’ Boolean logic is typically utilized. However, with this method, the original information cannot always be fully recovered, as shown in the last column of Figure 2(a) for an encrypted image of the character ‘1’ [15], [20]. To address these issues, a modified Boolean logic of ‘BITXOR’ is employed to the encryption scheme, where the Boolean rules are as follows: 1 ⊕ 1 = 0, 1 ⊕ 0 = 1, 0 ⊕ 1 = 1, and 0 ⊕ 0 = 0. In this algorithm, black pixels are encoded with the same sub-blocks, while white pixels are encoded with complementary sub-blocks, allowing each encoded pixel to fully recover the original information, as shown in the second last column of Figure 2(a). We performed numerical simulations to validate the decryption process. Figure 2(b) shows the decryption results of the encrypted images of characters ‘1’ and ‘2’ obtained through numerical simulation using the private key. The simulation results indicate complete decryption and recovery of the initial information.

## 3 Metasurface design

To enhance the capacity and security of optical encryption, we introduce a polarization-dependent multi-channel metasurface-based encryption scheme. Traditional polarization-dependent encryption schemes typically rely on orthogonal polarization states, which inherently limit the number of encryption channels and the complexity of the encryption process. Additionally, inter-channel crosstalk poses a significant challenge to ensuring the security and reliability of encryption systems, particularly as the number of polarization-dependent channels increases. To address these limitations, we developed a global optimization method across the entire metasurface that enables the design of multiple independent polarization-dependent channels with minimal crosstalk.

**Figure 3:**
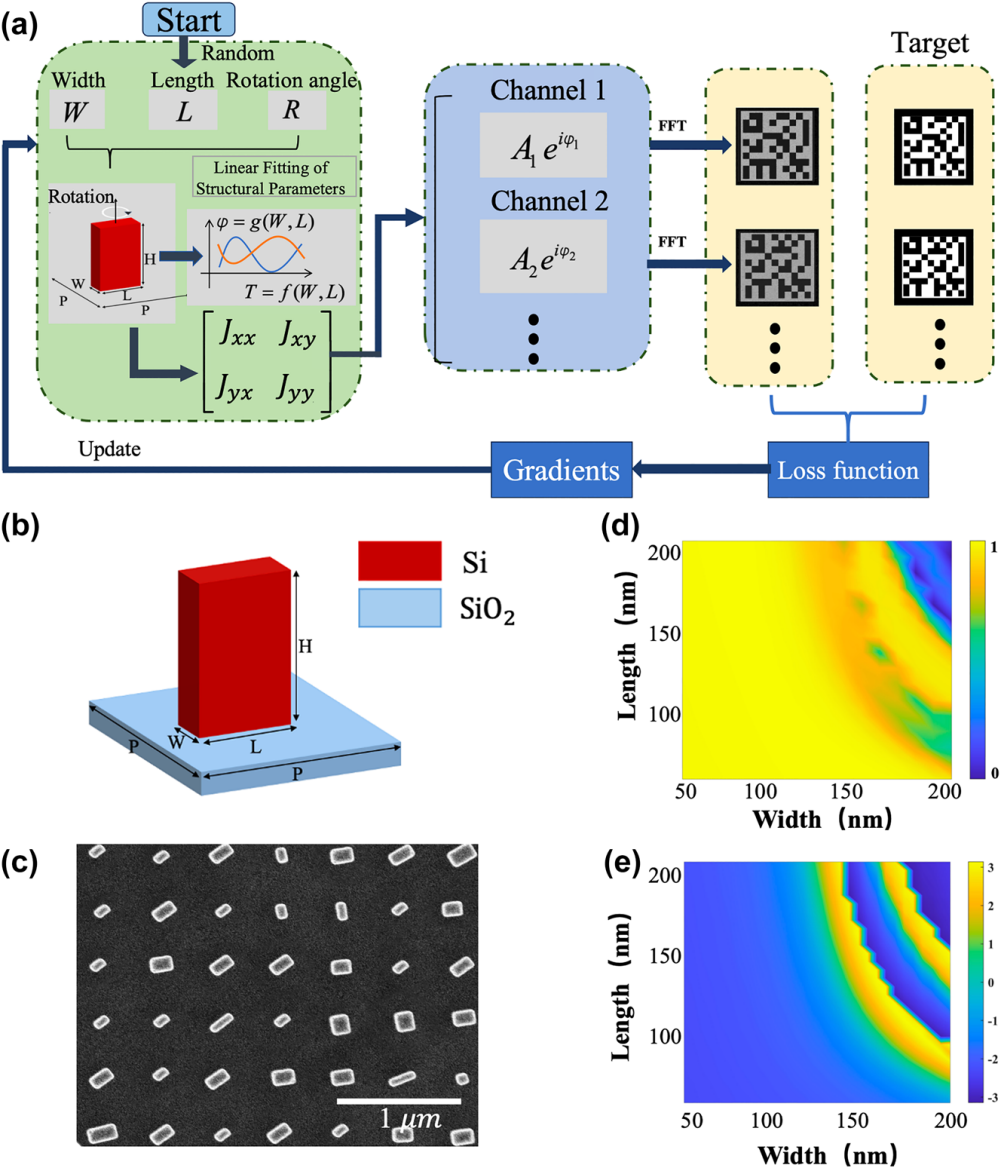
Design of polarization-dependent multi-channel metasurfaces. (a) The workflow of the optimization methodology. (b) Schematic of the unit cell structure. The period of the unit cell is *P* = 500 nm and the height is *H* = 500 nm. (c) SEM image of the metasurface. (d) and (e) Simulated transmission and phase delay for different nanorod dimensions.

The optimization workflow is depicted in Figure 3(a). The process begins with constructing a meta-atom library. The metasurface is based on silicon nanorods on SiO_2_ substrate as shown in Figure 3(b), with each unit cell having a periodicity of 500 nm. Figure 3(d) and (e) present the response maps of transmittance *T*_*x*_ and phase *ς*_*x*_ with respect to *x*-polarized light. These maps are acquired via finite-difference time-domain (FDTD) simulations conducted under a periodic boundary condition. In this simulation, the length (*L*) and width (*W*) of the structural parameters span from 50 nm to 200 nm, and the nanorods are set without any rotation angle. It is important to note that matrices *T*_*x*_ and *T*_*y*_ have a transpose relationship, and the same holds true for *ς*_*x*_ and *ς*_*y*_. The Jones matrix of each unit cell can be written as:

(1) $$\begin{array}{ll} & \left[\begin{array}{cc} J_{xx} & J_{xy}\\ J_{yx} & J_{yy} \end{array}\right]\\ & \;=\left[\begin{array}{cc} E_{x0}\cos^{2}\varphi +E_{y0}\text{si}{\text{n}}^{2}\varphi & \frac{1}{2}\left(E_{x0}-E_{y0}\right)\text{sin}2\varphi \\ \frac{1}{2}\left(E_{x0}-E_{y0}\right)\text{sin}2\varphi & E_{x0}\sin^{2}\varphi +E_{y0}\text{co}{\text{s}}^{2}\varphi \end{array}\right] \end{array}$$

where *ϕ* is the rotation angle of the nanorod, $E_{x0}=T_{x}{\text{e}}^{\text{i}\varsigma_{x}}$ and $E_{y0}=T_{y}{\text{e}}^{\text{i}\varsigma_{y}}$ represent the complex transmission coefficients for *x*-polarized light and *y*-polarized light of the nanorods, respectively. That is, the complex transmission coefficient of a nanorod for one polarization channel can be written as:

(2) $$E_{\theta }=\left[\begin{array}{cc} \cos\theta_{2} & \sin\theta_{2} \end{array}\right]\left[\begin{array}{cc} J_{xx} & J_{xy}\\ J_{yx} & J_{yy} \end{array}\right]\left[\begin{array}{c} \cos\theta_{1}\\ \sin\theta_{1} \end{array}\right]$$

where *θ*_1_ and *θ*_2_ are the angles of the incident polarization and the analyzing polarization, respectively. To maximize energy efficiency, both the incident and analyzing polarizations are aligned at the same angle *θ* [64], i.e. *θ*_1_ = *θ*_2_ = *θ*. The polarization-dependent complex amplitudes of distinct polarization channels are respectively associated with different values of *θ*. This approach can also be used to design multi-channel metasurfaces with different incident and analyzing polarization angles, further enhancing the number of channels and flexibility.

To transform the physical design problem into a differentiable mathematical problem for continuous gradient descent optimization, we perform a polynomial fitting to *E*_*x*0_ and *E*_*y*0_ with respect to the length (*L*) and width (*W*) of the nanorods. This results in a continuous function for the transmission coefficients to the nanorods with minimum errors. During the optimization process, the length, width, and rotation angle of the nanorods are iteratively optimized.

The optimization starts with initializing the metasurface array with random geometric parameters. We reconstructed polarization-dependent images via Fresnel diffraction. Next, the derivatives of the loss function corresponding to multiple images are calculated with respect to each meta-atom’s geometries. This establishes a direct relationship between the metasurface structural parameters and the output reconstructed images. Subsequently, we iteratively refine the design using the Adam optimizer in Python.

The loss function in the optimization process is specifically tailored for VSS encryption, focusing on three key factors: maximizing the correlation with the target image, minimizing inter-channel crosstalk (determined by the correlation of non-target regions), and optimizing overall efficiency. This ultimately achieves the uniformity and high contrast of the pixelated encoded image, which are crucial for reliable encoding and accurate information recovery. Furthermore, this specialized optimization strategy allows the entire process – from the basic structural parameters of the metasurface to the device functionality – to be completed within a single program, resulting in a flexible and efficient metasurface design. This method fully utilizes the degree of freedoms for polarization and phase manipulation of the nanostructures across the entire metasurface, enabling the realization of a greater number of independent polarization channels with minimal inter-channel crosstalk. It is worth mentioning that all target images are designed on the same propagation plane. If the target images are distributed across different planes or additional optical degrees of freedom (e.g., wavelength) are utilized, more channels can be easily realized.

Following this optimization methodology, we designed polarization-dependent multiplexed metasurfaces with different polarization encryption channels within the same plane. The designed metasurface consists of a 600 × 600 nanostructure array. Afterwards, the metasurface were fabricated using e-beam lithography, followed by lift-off, reactive ion etching (RIE), and hard mask removing processes (detailed in supplement). Figure 3(c) shows the scanning electron microscope (SEM) image of the fabricated metasurface.

## 4 Characterization and discussion

**Figure 4:**
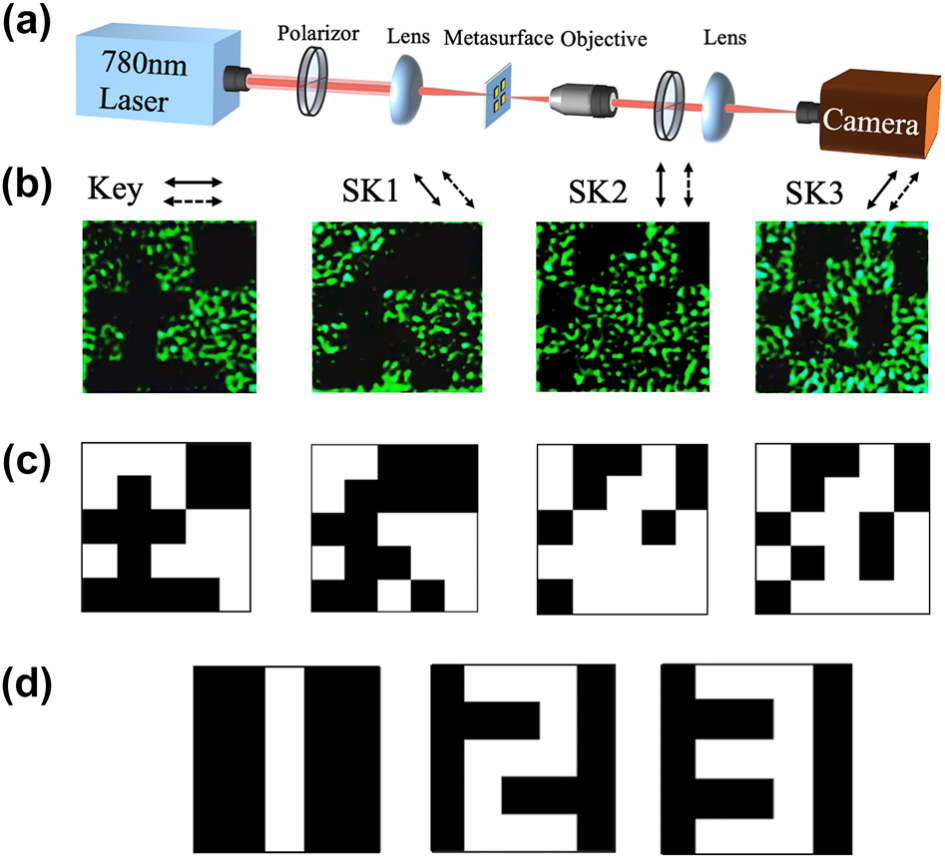
Experimental results for 4-channel encryption. (a) Experimental setup for the optical characterization. (b) and (c) The recorded holograms images of the polarization channels and the binarized images. The solid and dashed black arrows along with the images indicate the polarization direction of the incident light and the analyzing state setting of the output light. The polarization states have an equal interval is 45°. (d) The encrypted recovery results for the 4-channel encrypted images.

To verify the practicability of implementing pixelated encoding optically, we initially employed a straightforward non-expansion secret sharing scheme using four polarization channels (details in Supplementary Materials) [65]. The corresponding experimental setup is shown in Figure 4(a). A 780 nm laser beam is directed onto the metasurface sample after polarization adjustment using a polarizer. The transmitted light is collected with an objective lens (Olympus, 10×, NA 0.25) and analyzed by a second polarizer. The holographic images corresponding to the polarization channels are recorded by a CCD camera. These recorded holographic images serve as the basis for the VSS decryption process.

Figure 4(b)–(d) illustrates the experimental results for multiple polarization-dependent holography channels and encryption process. The color images in Figure 4(b) shows the encrypted holographic images corresponding to a four-polarization-channel metasurface recorded by the CCD camera. The results present four distinct images with little crosstalk between channels, indicating the effectiveness of the polarization multiplexing. Additionally, the images exhibit well-defined pixel edges, which are crucial for high-quality VSS encryption. The images are subsequently subjected to a binarization process to improve the visualization (details in Supplementary Materials). Finally, the key and ciphertext images are decrypted using the pre-designed “BITXOR” logic. As shown in Figure 4(d), the characters ‘1’, ‘2’ and ‘3’ are successfully decrypted with 100 % fidelities, confirming the effectiveness and accuracy of the proposed approach.

**Figure 5:**
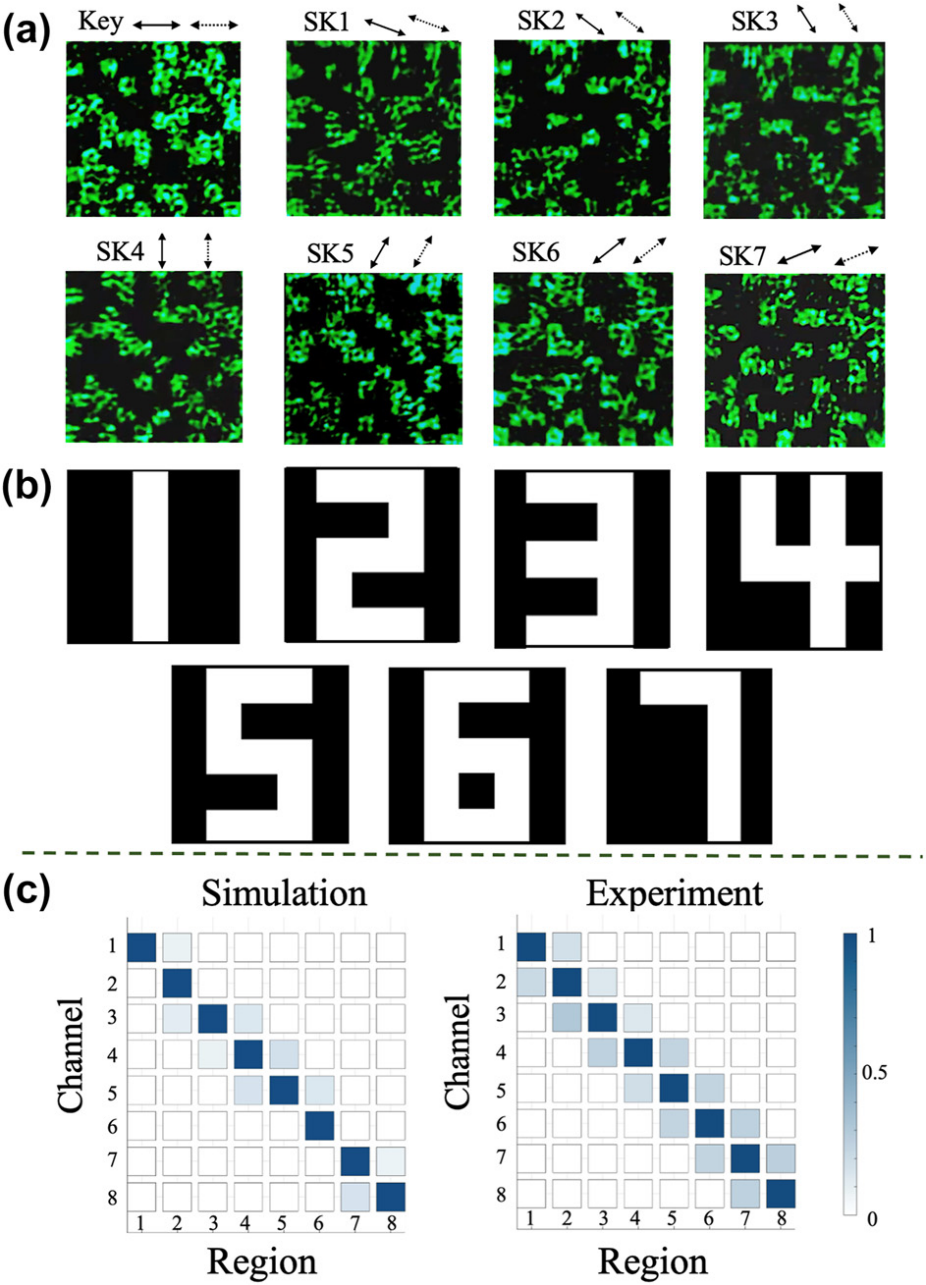
The experiment demonstration of VSS encryption based on 8 polarization-dependent channels. (a) The holograms of the 8 polarization channels. The polarization interval is 22.5°. (b) The encrypted recovery results. (c) The correlation coefficient matrix for the simulated and experimental results for the 8-channel encrypted images.

In order to verify the feasibility and reliability of the VSS scheme on multiple independent polarization channels, the scheme was extended to eight polarization channels for encryption, as shown in Figure 5(a). Each channel was evenly spaced at 22.5° polarization intervals with 10 × 10 encrypted pixels. One of the channels carried the private key, while the remaining seven channels encoded the encrypted images corresponding to the characters ‘1’ to ‘7’ using the enhanced encryption approach. By applying the same decoding process as the four-channel system, all the images were successfully recovered with 100 % accuracy, as shown in Figure 5(b). These results indicate the capability of the proposed method to securely transmit more complex information within a high-capacity scheme.

The crosstalk between the encryption images in different polarization channels is essential to the encryption performance. To evaluate the crosstalk between the polarization channels, we conduct a correlation measurement, using the following formula:

(3) $$\text{corr}\left(T,R\right)=\frac{\text{COV}\left(T,R\right)}{\sqrt{D\left(T\right)\cdot D\left(R\right)}}$$

Here, *T* and *R* represent the intensity distributions of the target image and the reconstructed image, respectively. As shown in Figure 5(c), the diagonal elements correspond to the target signal, and the off-diagonal elements indicate the level of crosstalk between different regions. In the simulation results, the correlation coefficients for off-diagonal elements remain below 0.20, whereas the diagonal elements reach values as high as 0.90, indicating that crosstalk in the target images is negligible. The experimental correlation matrix aligns closely with the simulation results. Although the diagonal elements from the experimental measurements are slightly lower than the simulation values, they still exceed 0.85, with most off-diagonal elements remaining below 0.25. Furthermore, we performed decryption attempts in the crosstalk regions to assess the effectiveness of the information encryption (detailed analysis provided in the Supplementary Information). These tests confirmed that no leakage of encrypted information occurred in the crosstalk regions, validating the robustness of the proposed encryption scheme.

Additionally, it is worth pointing out that all the VSS encryption channels in this work are designed to operate on the same propagation plane. The encryption channels can be increased by employing more image planes at different distances from the metasurface. This strategy can also be integrated to other degrees of freedom of light, such as wavelength [21], to further expand the encryption channels, thereby enhancing the information capacity and encryption security.

## 5 Conclusions

In summary, we have presented an advanced VSS encryption scheme based on multiple polarization-dependent channels facilitated by a metasurface. Through employing a global optimization methodology for nanostructure geometries across the entire metasurface, we successfully achieved eight independent polarization-dependent channels with minimal crosstalk, significantly enhancing both information capacity and encryption security. By encoding both the key and information into these channels through a modified VSS scheme incorporating ‘BITXOR’ logic, we successfully demonstrated the complete recovery of seven distinct plaintext images, representing a great improvement over conventional metasurface-based polarization-dependent holographic encryption methods. Our approach demonstrated superior performance by providing more encryption channels, reduced crosstalk, and significantly enhance the accuracy of the decrypted information. Furthermore, the demonstrated methodology is versatile and ready to be incorporated with other optical degrees of freedom, thereby offering a scalable platform for future developments in high-capacity secure communication, advanced information storage, and anti-counterfeiting technologies.

## Supplementary Material

Supplementary Material Details

## References

[1] Qu G. (2020). Reprogrammable meta-hologram for optical encryption. *Nat. Commun.*.

[2] Ouyang M. (2022). Optical encryption in spatial frequencies of light fields with metasurfaces. *Optica*.

[3] Zheng P. (2021). Metasurface-based key for computational imaging encryption. *Sci. Adv.*.

[4] Georgi P. (2021). Optical secret sharing with cascaded metasurface holography. *Sci. Adv.*.

[5] Chen W., Javidi B., Chen X. (2014). Advances in optical security systems. *Adv. Opt. Photonics*.

[6] Audhkhasi R., Lien M. R., Povinelli M. L. (2023). Experimental implementation of metasurfaces for secure multi-channel image encryption in the infrared. *Adv. Opt. Mater.*.

[7] Deng J. (2022). Metasurface-assisted optical encryption carrying camouflaged information. *Adv. Opt. Mater.*.

[8] Zhang F. (2023). Meta-optics empowered vector visual cryptography for high security and rapid decryption. *Nat. Commun.*.

[9] Guo X. (2022). Stokes meta-hologram toward optical cryptography. *Nat. Commun.*.

[10] Zhao R. (2018). Multichannel vectorial holographic display and encryption. *Light Sci. Appl.*.

[11] Hou J., Situ G. (2022). Image encryption using spatial nonlinear optics. *elight*.

[12] Jiao S., Zhou C., Shi Y., Zou W., Li X. (2019). Review on optical image hiding and watermarking techniques. *Opt. Laser Technol.*.

[13] Xu W., Xu H., Luo Y., Li T., Shi Y. S. (2016). Optical watermarking based on single-shot-ptychography encoding. *Opt. Express*.

[14] Zhang C. (2019). Compressive optical steganography via singlepixel imaging. *Opt. Express*.

[15] Li Z. (2021). Polarization-assisted visual secret sharing encryption in metasurface hologram. *Adv. Photonics Res.*.

[16] Naor M., Shamir A. (1995). Visual cryptography. *Advances in Cryptology – EUROCRYPT’94: Workshop on the Theory and Application of Cryptographic Techniques*.

[17] Shyu S. J., Huang S.-Y., Lee Y.-K., Wang R.-Z., Chen K. (2007). Sharing multiple secrets in visual cryptography. *Pattern Recogn.*.

[18] Zhou Z., Arce G. R., Di Crescenzo G. (2006). Halftone visual cryptography. *IEEE Trans. Image Process.*.

[19] Ateniese G., Blundo C., De Santis A., Stinson D. R. (2001). Extended capabilities for visual cryptography. *Theor. Comput. Sci.*.

[20] Li Z., Premaratne M., Zhu W. (2020). Advanced encryption method realized by secret shared phase encoding scheme using a multi-wavelength metasurface. *Nanophotonics*.

[21] Gu Z. (2024). Multi-wavelength metasurface empowered cryptography for heightened security and improved fidelity. *Laser Photonics Rev.*.

[22] Frese D., Wei Q., Wang Y., Huang L., Zentgraf T. (2019). Nonreciprocal asymmetric polarization encryption by layered plasmonic metasurfaces. *Nano Lett.*.

[23] Deng L. (2020). Malus-metasurface-assisted polarization multiplexing. *Light Sci. Appl.*.

[24] Yu N. (2011). Light propagation with phase discontinuities: generalized laws of reflection and refraction. *Science*.

[25] Xie X. (2021). Generalized Pancharatnam–Berry phase in rotationally symmetric meta-atoms. *Phys. Rev. Lett.*.

[26] Zhang X. (2019). Colorful metahologram with independently controlled images in transmission and reflection spaces. *Adv. Funct. Mater.*.

[27] Wang B. (2016). Visible-frequency dielectric metasurfaces for multiwavelength achromatic and highly dispersive holograms. *Nano Lett.*.

[28] Huang Y.-W. (2015). Aluminum plasmonic multicolor meta-hologram. *Nano Lett.*.

[29] Li J. (2021). Full-color enhanced second harmonic generation using rainbow trapping in ultrathin hyperbolic metamaterials. *Nat. Commun.*.

[30] Faraji-Dana M. (2018). Compact folded metasurface spectrometer. *Nat. Commun.*.

[31] Pu M. (2015). Catenary optics for achromatic generation of perfect optical angular momentum. *Sci. Adv.*.

[32] Ren H., Fang X., Jang J., Bürger J., Rho J., Maier S. A. (2020). Complex-amplitude metasurface-based orbital angular momentum holography in momentum space. *Nat. Nanotechnol.*.

[33] Deng M. (2024). Broadband angular spectrum differentiation using dielectric metasurfaces. *Nat. Commun.*.

[34] Ren H. (2019). Metasurface orbital angular momentum holography. *Nat. Commun.*.

[35] Iqbal S., Rajabalipanah H., Zhang L., Qiang X., Abdolali A., Cui T. J. (2020). Frequency-multiplexed pure-phase microwave meta-holograms using bi-spectral 2-bit coding metasurfaces. *Nanophotonics*.

[36] Yin Y. (2024). Multi-dimensional multiplexed metasurface holography by inverse design. *Adv. Mater.*.

[37] Wang J. (2024). Unlocking ultra-high holographic information capacity through nonorthogonal polarization multiplexing. *Nat. Commun.*.

[38] Wang D. (2024). Decimeter-depth and polarization addressable color 3D meta-holography. *Nat. Commun.*.

[39] Deng Z.-L., Wang Z.-Q., Li F.-J., Hu M.-X., Li X. (2022). Multi-freedom metasurface empowered vectorial holography. *Nanophotonics*.

[40] Deng Z. L. (2020). Full-color complex-amplitude vectorial holograms based on multi-freedom metasurfaces. *Adv. Funct. Mater.*.

[41] Ma W. (2022). Pushing the limits of functionality-multiplexing capability in metasurface design based on statistical machine learning. *Adv. Mater.*.

[42] Dong F., Chu W. (2019). Multichannel-independent information encoding with optical metasurfaces. *Adv. Mater.*.

[43] Chen S., Liu W., Li Z., Cheng H., Tian J. (2020). Metasurface-empowered optical multiplexing and multifunction. *Adv. Mater.*.

[44] Xiong B. (2023). Breaking the limitation of polarization multiplexing in optical metasurfaces with engineered noise. *Science*.

[45] Fan Y. (2024). Dual-channel quantum meta-hologram for display. *Adv. Photonics Nexus*.

[46] Shen Z., Lin X. (2023). A review of metasurface polarization devices. *Opt. Mater.*.

[47] Liu Z., Wang D., Gao H., Li M., Zhou H., Zhang C. (2023). Metasurface-enabled augmented reality display: a review. *Adv. Photonics*.

[48] Lee G.-Y. (2018). Metasurface eyepiece for augmented reality. *Nat. Commun.*.

[49] Gopakumar M. (2024). Full-colour 3D holographic augmented-reality displays with metasurface waveguides. *Nature*.

[50] Yang H., Jiang Y., Hu Y., Ou K., Duan H. (2022). Noninterleaved metasurface for full-polarization three-dimensional vectorial holography. *Laser Photonics Rev.*.

[51] Jiang Q., Jin G., Cao L. (2019). When metasurface meets hologram: principle and advances. *Adv. Opt. Photonics*.

[52] Zhao R. (2021). Polarization and holography recording in real- and k-space based on dielectric metasurface. *Adv. Funct. Mater.*.

[53] Song Q. (2020). Ptychography retrieval of fully polarized holograms from geometric-phase metasurfaces. *Nat. Commun.*.

[54] Chen W. T. (2014). High-efficiency broadband meta-hologram with polarization-controlled dual images. *Nano Lett.*.

[55] Zhao R. (2023). Stereo Jones matrix holography with longitudinal polarization transformation. *Laser Photonics Rev.*.

[56] H X. (2024). 3D multiview holographic display with wide field of view based on metasurface. *Adv. Opt. Mater.*.

[57] Zheng G., Mühlenbernd H., Kenney M., Li G., Zentgraf T., Zhang S. (2015). Metasurface holograms reaching 80% efficiency. *Nat. Nanotechnol.*.

[58] Deng Z.-L. (2018). Diatomic metasurface for vectorial holography. *Nano Lett.*.

[59] Khonina S. N., Butt M. A., Kazanskiy N. L. (2024). A review on reconfigurable metalenses revolutionizing flat optics. *Adv. Opt. Mater.*.

[60] Deng M. (2024). Dielectric metasurfaces for broadband phase-contrast relief-like imaging. *Nano Lett.*.

[61] Huang Z. (2023). High-resolution metalens imaging polarimetry. *Nano Lett.*.

[62] Yang H. (2023). Metasurface-empowered optical cryptography. *Mater. Today*.

[63] Yang H. (2023). Angular momentum holography via a minimalist metasurface for optical nested encryption. *Light Sci. Appl.*.

[64] Balthasar Mueller J., Rubin N. A., Devlin R. C., Groever B., Capasso F. (2017). Metasurface polarization optics: independent phase control of arbitrary orthogonal states of polarization. *Phys. Rev. Lett.*.

[65] Yang C. N. (2004). New visual secret sharing schemes using probabilistic method. *Pattern Recogn. Lett.*.

