# Supplementary material for: Enhanced optical encryption via polarization-dependent multi-channel metasurfaces
Source: Nanophotonics. 2025 Feb 13;14(4):495–502. doi: 10.1515/nanoph-2024-0746 (PMC11834057; doi:10.1515/nanoph-2024-0746)
Supplement: Supplementary file 1 — Supplementary Material Details [file j_nanoph-2024-0746_suppl_001.docx]

Supplementary Material for：Enhanced optical encryption via polarization-dependent multi-channel metasurfaces

Minghao Ning, Haozong Zhong, Zhen Gu, Ling-en Zhang, Ning Qu, Ding Jun*, Tao Li*, and Lin Li*

**Minghao Ning:** State Key Laboratory of Precision Spectroscopy, East China Normal University, Shanghai 200241, China; E-mail:52250920021@stu.ecnu.edu.cn;

**Haozong Zhong:** State Key Laboratory of Precision Spectroscopy, East China Normal University, Shanghai 200241, China; E-Mail:52210920043@stu.ecnu.edu.cn;

**Zhen Gu:** Shanghai Key Laboratory of Multidimensional Information Processing, Key Laboratory of Polar Materials and Devices, East China Normal University, Shanghai 200241, China ; E-Mail: [52254700016@stu.ecnu.edu.cn](mailto:52254700016@stu.ecnu.edu.cn);

**Ling-en Zhang:** State Key Laboratory of Precision Spectroscopy, East China Normal University, Shanghai 200241, China; E-mail: 52260920052@stu.ecnu.edu.cn

**Ning Qu:** State Key Laboratory of Precision Spectroscopy, East China Normal University, Shanghai 200241, China; E-mail: 51260920036@stu.ecnu.edu.cn

**Jun Ding:** Shanghai Key Laboratory of Multidimensional Information Processing, Key Laboratory of Polar Materials and Devices, East China Normal University, Shanghai 200241, China; Jun Ding, jding@ee.ecnu.edu.cn;

**Tao Li:** National Laboratory of Solid State Microstructures, College of Engineering and Applied Sciences, Nanjing University, Nanjing, 210093, China; taoli@nju.edu.cn;

**Lin Li**, State Key Laboratory of Precision Spectroscopy, School of Physics and Electronic Science, East China Normal University, Shanghai 200241, China; Collaborative Innovation Center of Extreme Optics, Shanxi University, Taiyuan 030006, China; lli@lps.ecnu.edu.cn

1. VSS Encoding method

The Visual Secret Sharing (VSS) scheme is a visual-based encryption method designed to divide a key or secret information into multiple shared parts (also referred to as "shares"). Each part appears random or meaningless, but when all the shared parts are combined, the original secret information can be restored. This encryption scheme is typically used for the secure transmission of images or visual information, particularly in cases where complex encryption algorithms are not desired. Depending on the subkey encoding method, VSS can be classified into non-expansion and pixel expansion schemes[1]. The core of the non-expansion method lies in how to recover the original image through specific decoding rules without increasing the image size. Its advantages include saving storage space and maintaining image quality, and it is often used for primary encryption where security requirements are not high. The pixel expansion scheme, another classic method in VSS, involves expanding each original pixel into multiple pixels. Its advantages lie in enhancing the security and complexity of the information. However, due to pixel expansion, the resolution of the recovered image may decrease. It is worth noting that in the VSS encryption algorithm, the combination of sub-keys is not limited to black and white pixels; as long as the sub-keys are randomly selected in a way that does not expose the initial information, the encryption remains secure[2].

The traditional (2,2) Visual Secret Sharing (VSS) scheme is a pixel-expansion encryption method. Each pixel of the original image is expanded into a 2×2 pixel array, containing binary sub-pixels of black and white. The binary distribution of each pixel is based on a predefined encryption function, ensuring that no single share independently reveals any information about the secret image. In this scheme, white pixels are encoded into two identical sub-image arrays, while black pixels are encoded into two complementary sub-image arrays, as shown in Fig. S1. A white pixel is composed of one of six random combination modes. Similarly, each option for black pixels has an equal probability. After re-encoding, each share is formed by a random combination of black and white sub-pixels, making a single share appear as random noise. This randomness prevents the deduction of the secret image from a single share. Only when both shares are combined do white pixels appear as mid-gray and black pixels as fully black, thereby reconstructing the original image.

The Visual Secret Sharing (VSS) scheme demonstrates robust security, as the interception of a single share reveals no meaningful information about the original secret image. However, as illustrated in Fig. S1, the use of identical sub-key selection with traditional "AND" Boolean logic for white pixels results in incomplete restoration of white pixels during decryption. In contrast, the enhanced VSS scheme updates the decryption logic to "BITXOR", enabling the complete recovery of both black and white pixels. As shown in Fig. S1(c), the decryption of the encoded digit "1" using both the enhanced and classical VSS encryption schemes reveals a significant drawback of the classical VSS scheme, where residual shared key artifacts remain in the decryption result, reducing the accuracy of the reconstruction.


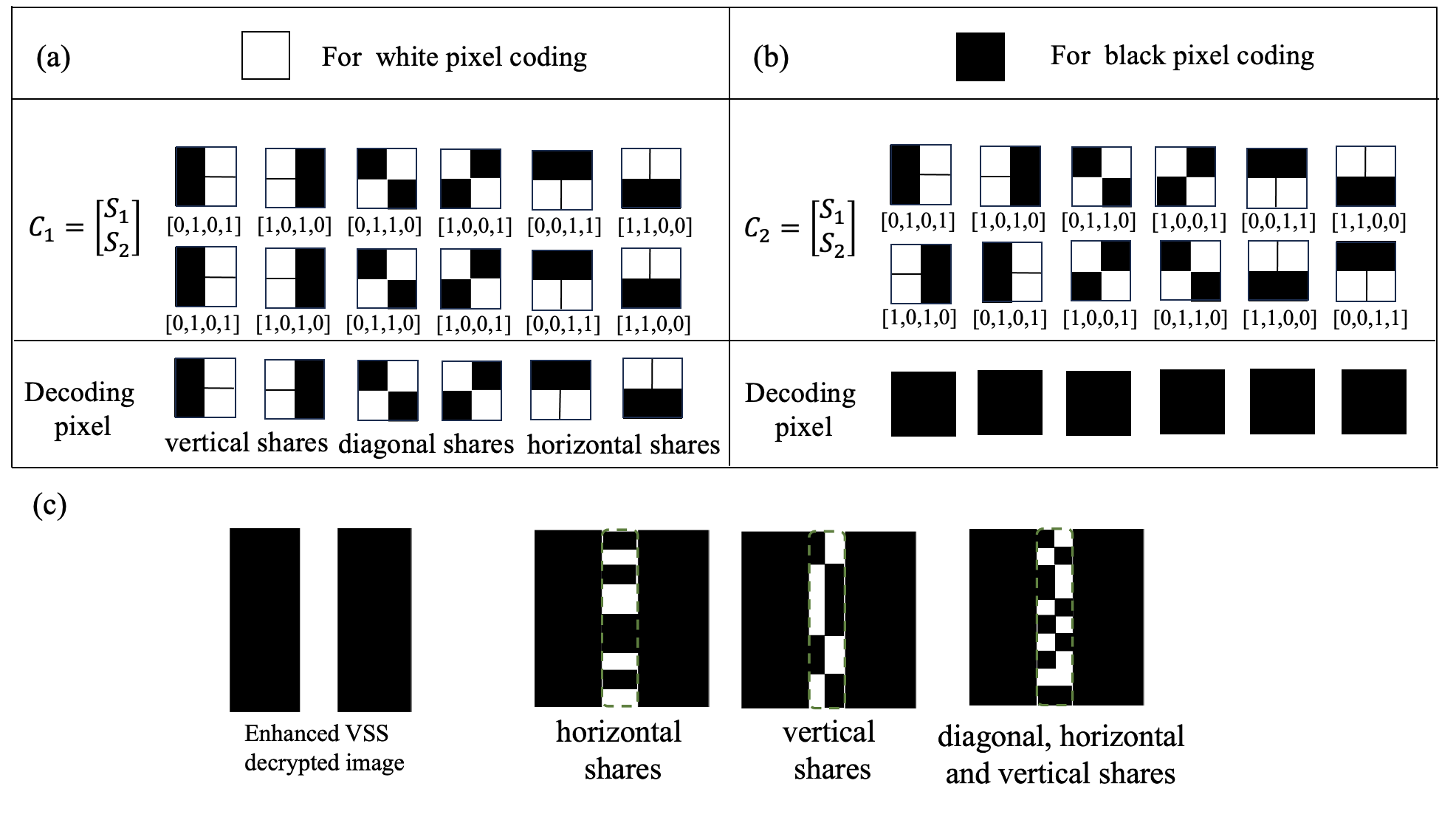


**Fig. S1:** Pixel encoding table for sharing information between black and white pixels in traditional scheme with AND decryption logic. (a) Encoding for white pixels. (b) Encoding for black pixels. (c) Decryption result of the enhanced VSS scheme (left) and the classical VSS scheme (right).

2. Fabrication method

The fabrication process of the microlens array involves a top-down nanofabrication approach. First, a silicon (Si) film is deposited onto a quartz substrate at 300°C using inductively coupled plasma chemical vapor deposition (ICP-CVD). After deposition, any organic residues on the surface are removed. Subsequently, a conductive positive electron beam resist (CSAR-6200) with a thickness of 120 nm is spin-coated onto the Si film. The patterns are defined in the resist using electron beam lithography (EBL, Elionix ELS-F125-G8). Then, a layer of chromium (Cr) is evaporated onto the sample, followed by a lift-off process, leaving a Cr hard mask on the Si layer for subsequent etching. The designed patterns are permanently etched into the Si layer using reactive ion etching (RIE) with a CHF₃ and O₂ gas mixture to precisely remove the layer. The remaining Cr hard mask is subsequently removed using a corrosive solution of ammonium cerium nitrate. Finally, any residual resist is removed by dry etching with O₂ plasma, a process known as descumming.

3. Dynamic scheme for secure information transmission

The encryption scheme with multiple independent polarization channels paves the way for high-capacity secure communication. To demonstrate the potential practicality of our multi-polarization channel metasurface for transmitting encrypted information, we propose a dynamic scheme for securely transmitting information and validate it through numerical simulations. In this scheme, the sender encrypts a sequence of phone numbers (e.g., "13087654321" as shown in the Fig. S2) using the multi-polarization channels of the metasurface. First, the sender encodes the digitized images of the encrypted phone numbers (SKs) into the metasurface across multiple polarization channels. Then, at a specific moment {$t_{m}\}$, the sender transmits three consecutive polarized signals with polarization $P_{m}$ as the starting signal of the encoded message. Next, the encrypted images are sequentially transmitted in time using multiple independent polarization channels. Once the transmission is complete, at time {$t_{m+s}$}, the sender transmits another set of three consecutive polarized signals with polarization $P_{m}$​ as the ending signal. The receiver uses a specialized receiving device to capture the encrypted images encoded in different polarizations over time. By applying the decryption logic, the receiver recovers the transmitted phone number. Finally, the decrypted phone number is successfully retrieved. Furthermore, as the size of the metasurface increases and more degrees of freedom become available, the number of transmission channels can be expanded through optimization algorithms, enabling the secure and efficient transmission of more complex information.


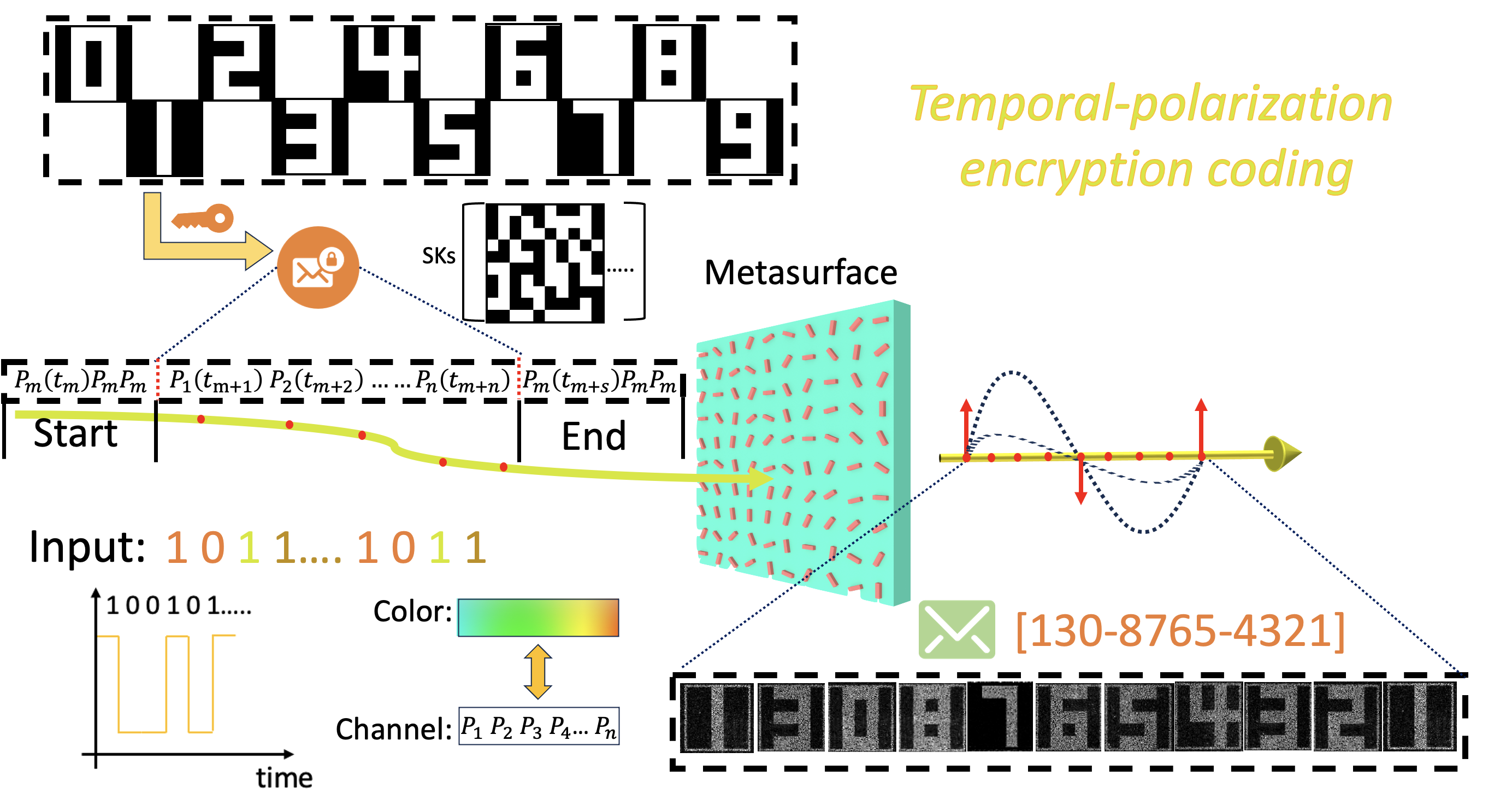


**Fig. S2**: A schematic diagram of the dynamic transmission process for encrypted information using the temporal-polarization encoding scheme.

4. Analysis of encryption security

In previous studies, the number of polarization channels has been limited by the tunable parameters within the Jones matrix of metasurfaces. When the number of independent polarization channels exceeds two, unavoidable crosstalk arises between the non-orthogonal polarization channels, which is unsatisfactory for holographic display applications. To achieve low-crosstalk multi-channel displays based on metasurfaces, some approaches have utilized additional hardware devices to introduce more degrees of freedom for channel control, while other studies have attempted to distribute holograms for different channels across separate propagation planes to reduce the impact of crosstalk. However, these multi-channel methods only enable corresponding channel displays, and significant crosstalk between different channels persists, preventing precise encryption and decryption of encoded pixels as achieved in the proposed scheme.

To address this limitation, we propose an inverse optimization method that explicitly incorporates crosstalk minimization, enabling independent encoding for non-orthogonal polarization channels within the same propagation plane. Furthermore, to evaluate the potential information leakage risks from crosstalk regions, we decrypted the non-encrypted regions under the target polarization channel, as shown in Figure S3.

During the averaging and binarization of the holograms in non-target regions, we reasonably deduced that when the ratio of black to white pixels approaches 1:1, the results are closest to the original subkeys (SKs). However, upon attempting to decrypt these regions, we found that the original information could not be retrieved, indicating that information transmitted through one encrypted channel does not expose the encrypted information in other channels. This further validates the robustness and reliability of the proposed encryption scheme.


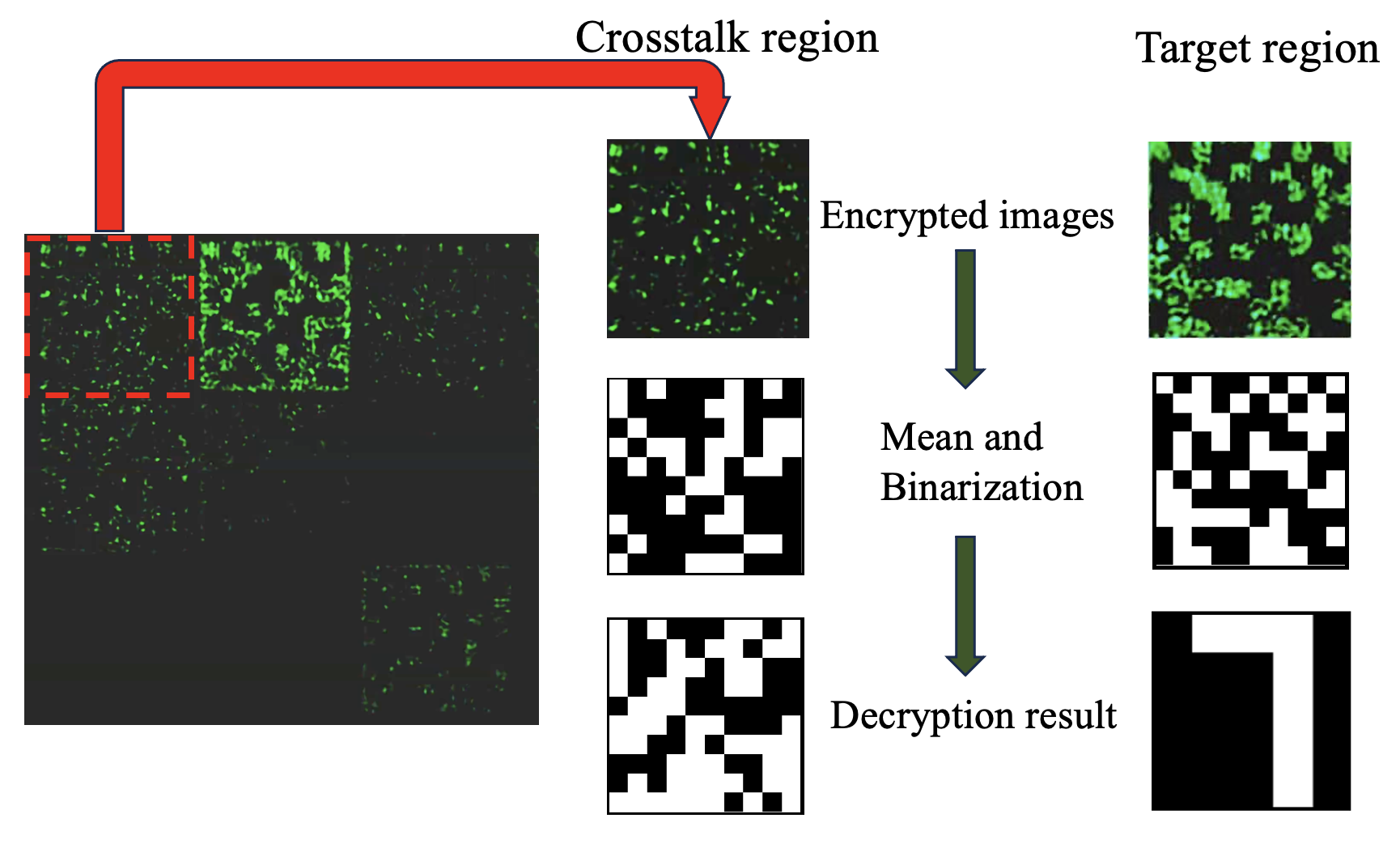


**Fig. S3:** Decryption of encrypted information from non-target channel and target channel under specific polarization analyzation.

To evaluate the security level of the polarization channels for encryption, we attempted to decrypt the encoded information by varying the polarization analysis angles with numerical calculation. As shown in Figure. S4, in our design, eight polarization channels were encoded at intervals of 22.5°, ranging from 0° to 157.5°. Taking channel 4 as an example, we found that complete recovery of the encrypted information could only be achieved within a polarization angle range of approximately 8°; outside this range, some pixels begin to reconstruct incorrectly (e.g., the decryption of the digit "3" outlined in the dashed circle).

Furthermore, we conducted numerical simulations for different polarization angle configurations, reducing the polarization angle interval between adjacent channels from 22.5° to 10°, while maintain eight independent channels. By decrypting the information under various polarization angles, we observed that as the angular interval between adjacent encoded polarization channels decreases, the corresponding decryption range also narrows (approximately 4° for a polarization angle interval of 10°). This means that the eight independent channels can only be successfully decrypted under precise polarization settings, with an accuracy of about ±2°.

This result indicates that higher precision in polarization angles is required to successfully decrypt the target polarization channel as the number of independent polarization channels increases or as the polarization angle intervals decrease, thereby enhancing the security of the encoded information.


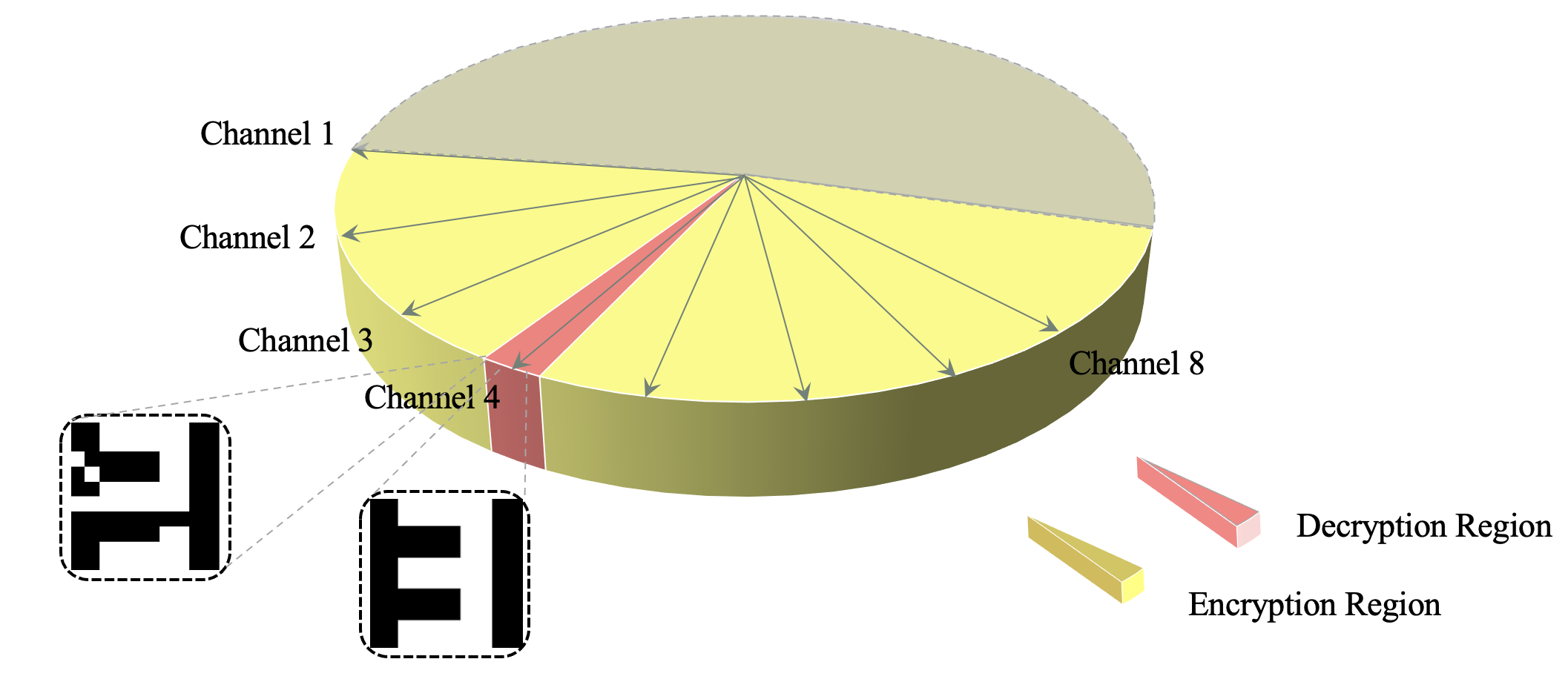


**Fig.S4:** Analysis of decoding encoded channels with different polarizations.

In summary, in our optimization algorithm, the polarization intervals between encoding channels can be arbitrarily set to achieve information encoding. Moreover, as the angular interval between adjacent polarization channels decreases, the decryption process demands higher precision in polarization angles, which further improves the security level of the encryption system.

**5.** **Discussion on the contrast of the experimental holography image**

In the VSS encryption scheme, the key factors for the pixelated images are uniformity and contrast. To evaluate the encrypted imaging performance, we analysed the cross-sectional intensity from the third image in Figure S5(b). The contrast between black and white pixels exceeds 4:1, ensuring easy differentiation between black and white encoding. Furthermore, the intensity within the encoded region is highly uniform, which facilitates decoding. While some relative noise points are present, they do not affect the overall pixel encoding resolution. In addition, these noise points can be reduced through mean operation, as shown in Figure S5(c). Based on this result, black and white pixel differentiation can be effectively achieved by setting a general threshold within the normalized intensity range of approximately 0.3–0.6. In the experiment, we set the normalized intensity threshold as 0.4. That is the pixel will be set as ‘1’ (white) when the intensity is greater than 0.4, otherwise, it will be set as ‘0’ (black).


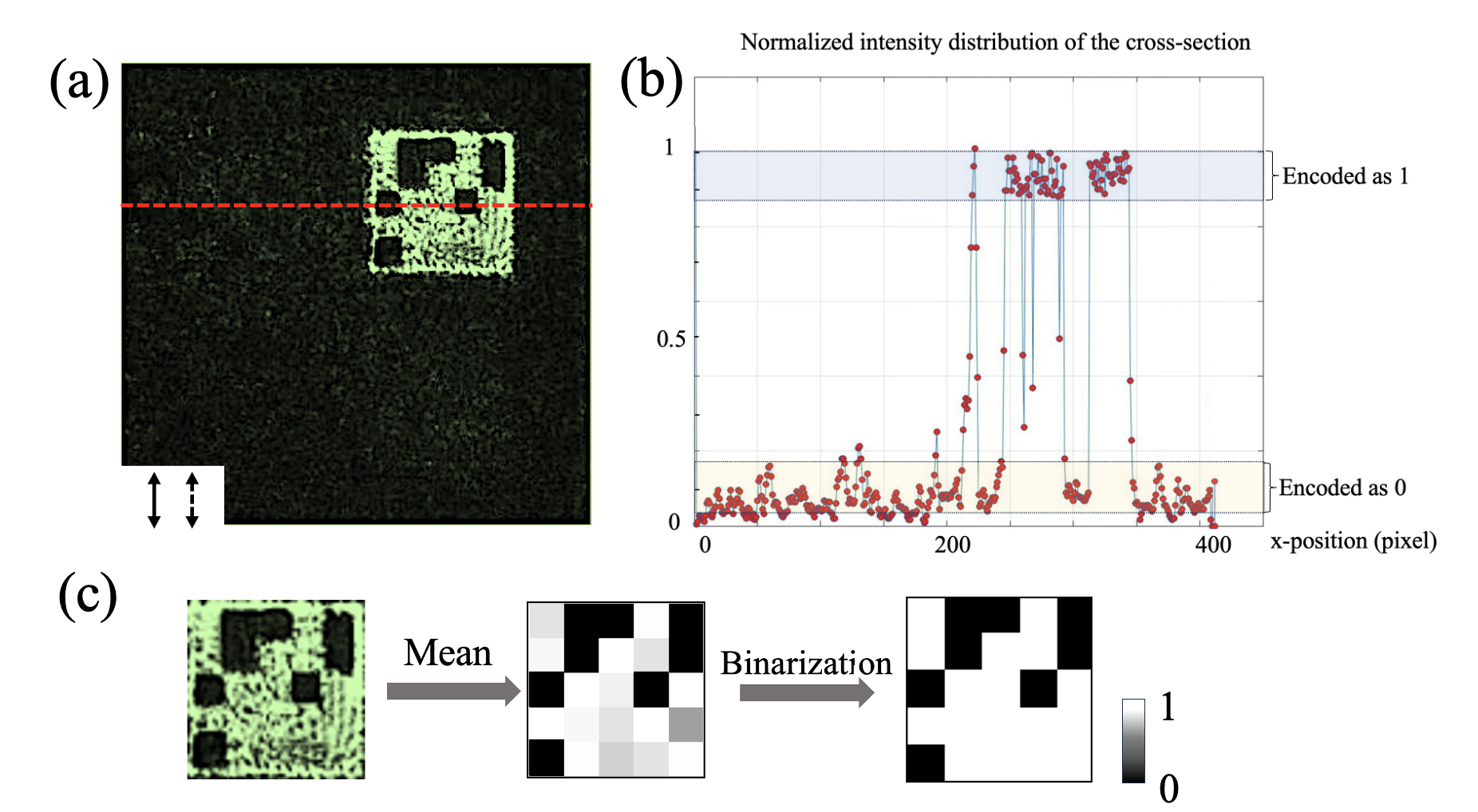


**Fig. S5:** Cross-sectional intensity distribution (with the horizontal axis $x$ representing spatial position).

1. C. N. Yang, "New visual secret sharing schemes using probabilistic method," Pattern Recognition Letters, vol. 25, no. 4, pp. 481-494, 2004.
2. Naor, M., & Shamir, A. ," Visual cryptography". Advances in Cryptology—EUROCRYPT'94: Workshop on the Theory and Application of Cryptographic Techniques Perugia, Italy, May 9–12, 1994 Proceedings, 13, 1-12. Springer Berlin Heidelberg, 1995.
3. B. Xiong, *et al.,* "Breaking the limitation of polarization multiplexing in optical metasurfaces with engineered noise," *Science*, vol. 379, pp. 294-299, 2023.
